# Smartphone Apps for Schizophrenia: A Systematic Review

**DOI:** 10.2196/mhealth.4930

**Published:** 2015-11-06

**Authors:** Joseph Firth, John Torous

**Affiliations:** ^1^ Institute of Brain, Behaviour and Mental Health University of Manchester Machester United Kingdom; ^2^ Brigham and Women's Hospital Department of Psychiatry Harvard Medical School Boston, MA United States; ^3^ Beth Israel Deaconess Medical Center Department of Psychiatry Harvard Medical School Boston, MA United States

**Keywords:** eHealth, fitness, mHealth, psychosis, schizophrenia, smartphones, technology, wearables

## Abstract

**Background:**

There is increasing interest in using mobile technologies such as smartphones for improving the care of patients with schizophrenia. However, less is known about the current clinical evidence for the feasibility and effectiveness of smartphone apps in this population.

**Objective:**

To review the published literature of smartphone apps applied for the care of patients with schizophrenia and other psychotic disorders.

**Methods:**

An electronic database search of Ovid MEDLINE, the Cochrane Central Register of Controlled Trials, Health Technology Assessment Database, Allied and Complementary Medicine, Health and Psychosocial Instruments, PsycINFO, and Embase was conducted on May 24, 2015. All eligible studies were systematically reviewed, and proportional meta-analyses were applied to pooled data on recruitment, retention, and adherence to examine the overall feasibility of smartphone interventions for schizophrenia.

**Results:**

Our search produced 226 results from which 7 eligible articles were identified, reporting on 5 studies of smartphone apps for patients with schizophrenia. All examined feasibility, and one assessed the preliminary efficacy of a smartphone intervention for schizophrenia. Study lengths varied between 6 and 130 days. Overall retention was 92% (95% CI 82-98%). Participants consistently used the smartphone apps on more than 85% of days during the study period, averaging 3.95 interactions per person per day. Furthermore, participants responded to 71.9% of automated prompts (95% CI 65.7-77.8%). Participants reported a range of potential benefits from the various interventions, and user experience was largely positive.

**Conclusions:**

Although small, the current published literature demonstrates strong evidence for the feasibility of using smartphones to enhance the care of people with schizophrenia. High rates of engagement and satisfaction with a broad range of apps suggest the nascent potential of this mobile technology. However, there remains limited data on the efficacy of such interventions.

## Introduction

The growing prevalence of smartphone technology has created increasing interest in mHealth across all areas of medicine. With global smartphone ownership already at 25% [1], and nearly 65% in countries like the United States [2], these advanced mobile phones offer a novel platform to expand the impact and reach of traditional health care services. One area that stands to potentially benefit from mHealth is the behavioral health sciences, such as psychiatry, given that smartphones could serve as proxies for capturing digital phenotypes of behaviors [3], and providing real-time psychological support.

Despite recent advances in research and practice, schizophrenia (and other psychotic illnesses) is still associated with high chronicity, disability, and global burden of disease [4]. Nonetheless, smartphone technologies may be able to assist with the diagnosis, monitoring, and treatment of psychotic illnesses, providing novel and cost-effective interventions with potentially global reach. However, little is actually known about the clinical realities of smartphones in the care of patients with psychotic illnesses.

Psychiatric diagnoses, especially psychosis and schizophrenia, already carry tremendous stigma [5,6], which exists even on digital platforms like Twitter [7]. Although there are limited data on smartphone usage among individuals with schizophrenia, it has been suggested that symptoms such as paranoia, disorganization, and cognitive impairment may limit the feasibility of technology-based interventions for this patient group. Patients with schizophrenia may therefore face a double stigma when approaching smartphones, from not only the nature of their illness, but also attitudes regarding their capacities to engage with such technologies [8]. However, preliminary evidence indicates that patients with schizophrenia likely may own and use technology in ways similar to the general population [9,10].

Along with the global population, evidence suggests that patients with schizophrenia increasingly own mobile phone and smartphones. A recent meta-analysis suggests that the overall mobile phone ownership among patients with psychosis is high at 81.4% for those surveyed in the last 2 years, although the study does not report specifically on smartphones [11]. A 2014 survey study of patients at a state-run mental health clinic in Boston, Massachusetts, found that over one-third of patients already have their own smartphones [12]. An earlier study of 1592 patients with severe mental illnesses, conducted in 2013, noted that 81% of those who owned a mobile phone were amenable toward receiving technology-enabled mental health services via their mobile phone [13]. Ownership appears to be particularly high among younger people in the earlier stages of illness, with around 69% of first-episode psychosis patients owning Internet-enabled mobile devices [9]. Global predictions of smartphone ownership suggest that these devices will become increasingly prevalent as barriers to ownership continue to diminish, and will soon replace most traditional mobile phones [14]. Thus, it is logical to assume that with time, smartphone ownership and use will become even more common in patients with schizophrenia. From the ability to identify at-risk individuals, track symptoms in outpatients, prevent relapse, encourage medication adherence, offer on-the-go support, and increase access to services, much has already been speculated about the potential of smartphone technologies to support various aspects of care for patients with schizophrenia [15,16]. In addition, patients with schizophrenia are increasingly being offered apps for self-monitoring and self-management purposes. For instance, the National Alliance of Mental Illness recently released its own app, “AIR,” [17] and there are numerous other apps for managing schizophrenia already available for download on iTunes and Android marketplaces.

Despite the increased interest and opportunities for using smartphone apps to improve outcomes in individuals with schizophrenia, there is currently limited empirical evidence to support their implementation into clinical practice. Studies that have trialed the utility of smartphones in the treatment of other mental illnesses have had mixed success, with some appearing beneficial [16], others failing to have significant impact [18], and some even indicating potential harm in certain patient groups [19]. Although academic research started examining the utility of smartphone apps in the care of schizophrenia many years ago [20], the literature has yet to be systematically examined.

Understanding the existing literature on the role of smartphones in schizophrenia will increase our understanding of what has been learnt so far, where the potential of this technology may be best realized in future, and where the speculations may actually be more theoretical rather than practical at this point. Thus, in this paper we aim to systematically review all existing studies of smartphone app trials in people with psychotic disorders and schizophrenia, and explore the current evidence base for their potential to impact on clinical care.

## Methods

### Eligibility Criteria and Study Selection

Only original, peer-reviewed, English research articles were included in the review. We aimed to include all published studies of smartphone apps used for improving care in schizophrenia. Thus, we included any studies that reported on any quantitative outcomes of a smartphone-based intervention among patients with schizophrenia. We defined a smartphone as a mobile phone with Internet connectivity and the ability to download and run third-party software apps available from a commercial marketplace. Studies that included patients with unspecified psychotic disorders, “severe mental illness,” or “serious mental disorders” were also eligible for review, provided that it could be confirmed that a portion of the sample studied did have a diagnosis of schizophrenia.

An electronic database search of Ovid MEDLINE, the Cochrane Central Register of Controlled Trials, Health Technology Assessment Database, Allied and Complementary Medicine (AMED), Health and Psychosocial Instruments, PsycINFO, and Embase was conducted on May 24, 2015, using the following keyword search algorithm: (“smartphone*” or “mobile phone*” or “cell phone” or “iPhone” or “mobile app*” or “phone app*”) AND (“psychiatric disorder*” or “severe mental” or “serious mental” or “psychosis” “psychotic” or “schizo*”).

Titles and abstracts of search results were screened by both authors using the aforementioned criteria. For any articles that were not excluded at this stage, the full text was retrieved and assessed for eligibility. The reference and citation lists of eligible articles were also searched to identify further studies. For any disagreements arising between the authors, discussion on that study was conducted by the authors until a consensus was reached. All articles matching our criteria were reviewed in full.

### Data Extraction and Analysis

To examine the overall feasibility of smartphone apps for schizophrenia, rates of recruitment, retention, and adherence were extracted from each study. “Recruitment rates” were calculated as number of patients referred divided by number consenting to take part. “Retention” was defined as the proportion of participants who remained in the study for the entire duration and completed follow-up assessments (where applicable). “Adherence” was examined in regards to the number of days using the smartphone app, number of uses-per-day, and also the response rate to automated prompts. These feasibility data from each individual study were pooled using proportional meta-analysis in StatsDirect 2.7 [21]. A DerSimonian-Laird random-effects model was applied to all analyses to account for heterogeneity between studies [22]. Between-study variance was assessed with Cochran’s Q and indexed as *I* ^2^, which estimates the amount of variance caused by interstudy heterogeneity rather than by chance. Given that only 1 study reported efficacy data, it was not possible to examine the effectiveness of smartphone interventions for schizophrenia with meta-analytic methods.

## Results

### Search Findings

**Figure 1:**
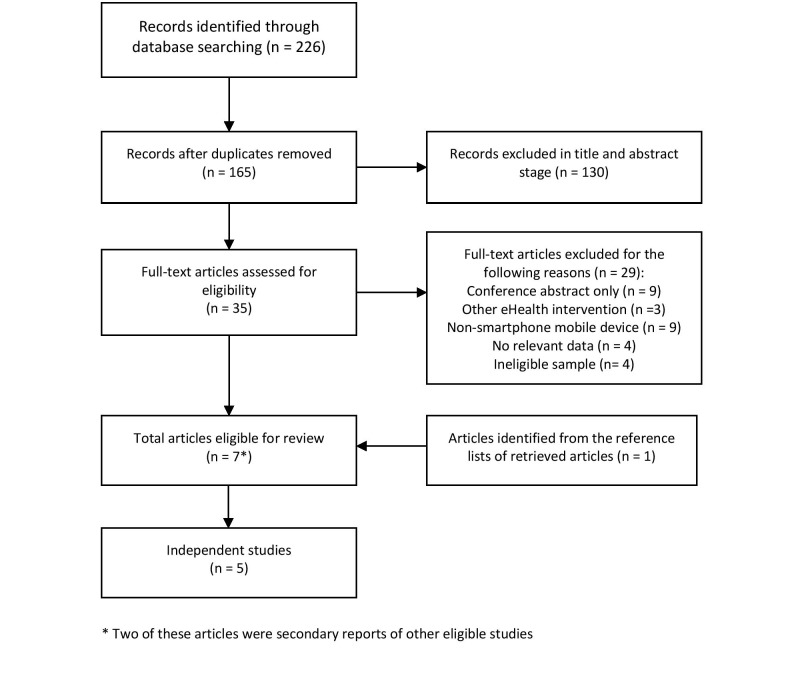
Study selection process.

The study selection process is detailed in Figure 1. The search strategy returned 226 results, providing 165 unique citations after duplicates were removed. Of these, 6 studies met eligibility criteria. A further study was identified from reviewing the references of the retrieved papers. The 7 eligible studies identified reported data from 5 independent trials, which were reviewed in full. Table 1 provides summary information from all included studies. However, there was substantial heterogeneity across studies, due to the fact that each app was unique. Thus, individual results are presented in the context of each individual study below (see Multimedia Appendix for a complete description of the intervention presented in Table 1).

### Systematic Review of Studies

Palmier-Claus et al [23] was one of the first to examine the role of smartphones for schizophrenia. The authors assessed the ability to use smartphones as a platform to collect clinical metrics in real time [23]. The study piloted a smartphone app to deliver 12 items from the Positive and Negative Syndrome Scale (PANSS) and 2 from the Calgary Depression Scale (CDS) up to 6 times per day over 1 week in 36 patients: 12 with remitted schizophrenia/schizoaffective disorder, 12 with acute schizophrenia, and 12 who were identified as “at-risk” for schizophrenia. Across all patients, mean adherence with the app’s survey sessions was 72%, with 82% completing at least 33% of survey sessions. Of the 8 patients who failed to complete at least 33% of the surveys, 6 were from the acute schizophrenia group.

Furthermore, the study found that smartphone apps can be used to obtain clinically meaningful self-assessments of psychiatric symptoms. Comparing app-based psychiatric ratings with those obtained from traditional paper-and-pencil ratings for the PANSS and CDS, positive symptom scales and affective symptom scales showed moderate to strong correlations, whereas questions about social withdrawal, excitement, hostility, and cognitive disorganization showed nonsignificant correlations [23]. A later analysis of results from this study suggested that the relationship between hallucinations and self-injurious thoughts may be mediated by the degree of paranoia and that paranoia predicted greater levels of self-injurious thoughts on subsequent days [24].

Ainsworth et al [25] compared how patients with schizophrenia reported their symptoms with a smartphone app versus text-message reporting of symptoms. Twenty-four patients with schizophrenia partook in this randomized repeated-measures crossover study, with all participants using both the smartphone app and text-messaging systems for 6 days each, receiving 4 short surveys about their symptoms per day. Results showed that smartphone-based symptom monitoring was preferable to text-message symptom monitoring: participants completed app-based symptom assessments on average nearly 5 times faster than on the text-messaging platform. Furthermore, participants completed a significantly greater number of entries when using the native smartphone app than using the text-message interface (69% vs. 56%) [25]. Qualitative results noted that participants were most comfortable using their own personal devices to complete surveys and felt that reporting symptoms via mobile phone was not stigmatizing, regardless of whether it was text messaging based or app based [26].

The FOCUS trial [27] investigated the feasibility and preliminary efficacy of a smartphone app to support the self-management of mental health for patients with schizophrenia. Thirty-three patients with schizophrenia/schizoaffective disorder in community treatment programs were recruited for this 1-month study. The smartphone app, “FOCUS,” deployed real-time interventions to target medication adherence, social functioning, mood problems, auditory hallucinations, and sleep difficulties. Participants were asked to complete automatically prompted surveys 3 times per day. In response to participants’ input, the app would also offer a self-management intervention. Participants could also use the intervention content on demand, whenever they wanted. Across all participants, 87% felt the app helped them manage symptoms. Mean adherence was 86.5%. Interestingly, the majority of app usage (62.5%) was self-initiated by participants, rather than on demand to daily prompts.

Two further studies examined the utility of smartphones to increase physical activity in patients with serious mental illnesses, including schizophrenia along with other disorders such as bipolar and major depressive disorder. The first of these included 10 patients with serious mental illness (3 of whom had schizophrenia). The study assessed the feasibility and acceptability of a wearable fitness tracker (Fitbit), paired with a smartphone app, to facilitate participant engagement in a weight loss program over a study duration of 80-113 days. Participants were highly adherent to the Fitbit, with a mean rate of daily usage of 89% during the study period. The study presented qualitative feedback about participant’s experience with the Fitbit and iPhone app, which was positive overall. However, because data were not linked to individual participants’ diagnosis, it is difficult to make any generalizations about the study’s results for patients with schizophrenia [28].

Nevertheless, a secondary report of the same study [28] presented additional findings, showing that patients were satisfied with the program, and found that it helped them to reach their goals. Because this response was obtained from all participants, we can be certain that these particular findings do apply to those with schizophrenia. In addition, the study noted that none of the participants reported privacy concerns about using these mobile technologies [29].

Another small study investigated a smartphone app, WellWave, to promote physical activity, specifically walking, among patients with schizophrenia, bipolar disorder, and/or major depression [30]. The app encouraged users to be more active, and offered mobile surveys related to overall health. It also allowed them to text message study staff and to access reading and watch videos about recovery. Ten patients were recruited, 4 with schizophrenia. Over the 4-week study period, mean adherence to daily app usage was 94%, and engagement with automated prompts was 73%. However, only 39% were complaint with daily walks, which the app prompted. Nonetheless, 3/10 patients reported significant improvements in self-ratings of physical health after just 4 weeks of usage [30].

**Table 1:** Studies of smartphone apps for people with schizophrenia.^a^

Studies	Intervention	Duration (days)	Total (n)	Attrition	Adherence	User experience	Reported benefits
Ainsworth et al (2013) [25]	Android app “ClinTouch”	6	24 (24 with schizophrenia)	0/24	69% of all possible entries were completed 2.8 uses per day (mean average)	The app was rated as “pleasing” overall (scoring 3.7 on a 7-point scale). The app was not rated as “stressful” or “challenging” (scoring only 1.8 and 2.2 on 7-point scales)	Participants felt the app could help them or other service users (5.3 on a 7-point scale)
Ben Zeev et al (2014) [27]	Android app “FOCUS”	28	33 (33 with schizophrenia)	1/33 due to losing phone	Participants used FOCUS on 86.5% of days in the study 5.2 uses per day (mean average)	93.7% of participants satisfied with overall ease of use. Less than 20% found the app to be “awkward,” “complicated,” or “inconsistent.”	87.5% of participants felt that the app helped to manage symptoms. Paired samples *t* tests showed significant reductions in positive and negative symptoms and depression.
Macias et al (2015) [30]	iPhone and Android app ‘WellWave’	28	11 (4 with schizophrenia)	1/11 withdrew of own accord	Used on 94% of days 73 % response rate to prompts (3.54 per day) 70% of participants achieved ≥2 walks per week	100% of participants were satisfied with the app overall. Only criticisms were made, pertaining to color/sound preferences, and the study coming to an end.	Participants experienced both improved well-being (eg, put my head in a good place) and practical benefits (eg, Motivated me to get up and walk around the block).
Naslund et al (2015) [28] Aschbrenner et al (2015) [29]	iPhone app “PeerFIT”	80-133	10 (3 with schizophrenia)	1/10 withdrew due to medical reasons	Participants used activity monitors on 89% of days in the study	100% were “very satisfied” or “somewhat satisfied” with PeerFIT overall; 60% would recommend to a friend. Participants felt the devices were expensive for low-income individuals.	100% found the program helped them to reach their goals. Mean weight loss of 2.7 kg across all participants (*P*>.05).
Palmier-Claus et al (2012, 2014) [23,24]	Android app “ClinTouch”	7	44 (36 with schizophrenia)	8/44 due to noncompliance	72% of all possible entries were completed 4.4 uses per day (mean)	Not reported	Smartphone app provided clinically valid real-time measures of psychotic symptoms and affective state

^a^This is an abridged version of the table. Additional details about the intervention are presented in Multimedia Appendix 1.

### Feasibility Data Analysis

To determine the feasibility of smartphone apps among individuals with schizophrenia, we pooled data from all of the individual studies reviewed [23,25,27,28,30] using proportion meta-analysis with a random-effects model. All 5 studies reported on retention over the study period. The study periods ranged from 6 to 130 days and the total retention rate was 92% (95% CI 82-98%, n=122), with moderate heterogeneity between studies (Cochran’s *Q*=9.52, *P*=.05, *I* ^2^=58.8% [95% CI =0-82.3%]). For the 4 studies [23,25,27,28] that reported recruitment (n=208), the overall rate of referral to enrollment was 57% (95% CI 31-82%, Cochran’s *Q*=43.43, *P*<.01, *I* ^2^=93.1%), with only 26% of referred patients uninterested in participating (as the rest were either ineligible or not contacted).

Automated prompts were used in 4 studies, although only 3 of these reported the response rate [23,25,30]. Studies used 4-6 alerts per day, and the average response rate across 70 participants was 71.9% (95% CI 65.7-77.8%), with moderate heterogeneity between studies (Cochran *Q*=5.83, *P*=.054, *I* ^2^ =65.7 [95% CI 0-88.1%]). Further summary statistics were calculated for adherence variables that could not be analyzed using proportional meta-analytic methods. Four studies reported number of uses per day [23,25,27,30]. Weighted mean averages show that apps were interacted with, on average, 3.94 times per day during the study periods, ranging from 2.8 to 5.2 uses per day. Participants used the apps on 86.5-94% of days during the studies [27,28,30].

## Discussion

### Findings From Reviewed Studies

Although only a small number of studies have assessed smartphone apps for schizophrenia, with only 5 trials identified by our review, early results are encouraging. The existing literature shows that people with schizophrenia are willing and able to use smartphones to monitor their symptoms, engage in self-directed therapeutic interventions and increase their physical exercise. Rates of retention and adherence during the trials proved to be high, with 92% of participants remaining in the trials until the end, and interacting with the apps on approximately 86.5-94% of days in the study. These rates of adherence and engagement appear similar, and actually slightly higher, compared with mHealth interventions for other chronic conditions such as diabetes, cardiovascular diseases, and lung diseases [31].

Furthermore, no paper reported any adverse outcomes or cases of app use increasing paranoia or exacerbating the symptoms of schizophrenia. However, 1 participant in Ainsworth et al’s study [25] did withdrew from reporting her symptoms as she found that this was making her ruminative; however, it has to be noted at that time, this patient was in the text messaging arm and not in the smartphone arm of the study. Overall, the existing literature indicates high feasibility of smartphone apps for patients with schizophrenia and other psychotic disorders. Despite been typically regarded as a difficult population to engage with health services, our results suggest that these patients are as engaged, active, and adherent with smartphone apps as other patient populations, such as those with diabetes [32-34]. However, the results of our review also raise several important discussions points and questions regarding which smartphone interventions might be better for patients, the validity of smartphone data, clinical role of smartphones, and issues related to next steps for research and clinical psychiatry.

Just as schizophrenia is a complex disorder with diverse manifestations, smartphone app use among patients with schizophrenia is also complex. Results reported by Palmier-Claus et al [23] suggest that patients in the acute stages of illness may have more difficulty with app adherence than those in partial or full remission. In addition, the highest rates of app adherence were observed among the group of participants who were classified as “at-risk” for schizophrenia, due to showing early warning signs, but did not yet have the full diagnosis. Cross-sectional studies have also found that younger psychiatric patients, in the earlier stages of illness, have the highest rates of smartphone ownership and usage [8,9,12]. Considered together, this evidence suggests that “at-risk” or “first episode” populations may ultimately be the most willing and able to use smartphones for monitoring their symptoms and engaging in mental health self-management, to prevent further (or first) psychotic episodes. Nonetheless, even older patients may benefit from smartphone-based interventions. Macias et al [30] reported that the greatest improvements in self-reported physical health from the “WellWave” app were reported by participants over the age of 50.

Although smartphones have proven feasible for real-time symptom monitoring, there is now a need to assess the validity of these measures, comparing traditional clinical metrics of psychiatric symptoms with those obtained via smartphone apps. Palmier-Claus et al [23] noted that positive symptom and affective symptoms assessed via smartphone prompts held significant correlations with traditional measures. However, weaker, nonsignificant correlations were observed for questions about social withdrawal, excitement, hostility, and cognitive disorganization. Potential reasons why smartphone self-assessments of these particular areas may differ from clinical metrics include recall bias, fear of judgment or consequences (eg, involuntary hospitalization), or even a genuine alteration in patients’ state when interacting with technology, rather than a human being. However, further research is needed to better understand this important issue. Furthermore, the capacity of smartphones to collect “passive data” (from location, general phone usage, or even wearables) may eventually eclipse the utility of smartphone-aided self-report. In any case, it is important to keep in mind that smartphone-collected data is a new metric in itself, which will require clinical validation and reliability evaluation for further novel apps.

The immediate clinical role of smartphone apps appears promising. Results reported by Ben-Zeev et al [27] suggested that patients with schizophrenia were not only compliant with an app that assisted mental health self-management, but actually found the app so useful that they used it at a much higher rate than required by the study. Results of two further studies that used smartphones apps to support exercise programs for people with serious mental illnesses (including schizophrenia) also had positive feedback, high levels of engagement and appeared to effectively increase physical activity [28-30]. Given that the prevalence of obesity and cardiometabolic disorders is double among people with psychotic disorders [35], smartphone apps may offer a new tool for engaging patients in regular exercise to attenuate cardiometabolic risk. This may also confer additional psychological benefits, as moderate-to-vigorous exercise can significantly reduce psychiatric symptoms among people with schizophrenia [36]. Thus, the clinical potential for smartphone apps in psychotic illnesses appears broad, and likely will continue to expand.

### Implications for Future Research

Considering the goal of this review was to explore the current evidence base for the potential of apps to impact clinical care, it must be acknowledged that there is currently much still unknown. While this review was able to identify and summarize information on acceptability, feasibility, satisfaction, and engagement, data on efficacy and clinical utility are still lacking. Examining these outcomes of smartphone apps for schizophrenia is an important target for future studies.

The result that participants feel more comfortable using their own personal devices, instead of study phones [26], makes intuitive sense, and suggests a means to lower costs and increase adherence in future research studies. While text messaging remains an important means of communication in this digital age, smartphone-based interventions are quicker, and result in higher rates of adherence than the equivalent text-messaging interventions and thus a better platform for research studies [25]. The ability of smartphones to collect real-time symptom data creates the opportunity to answer questions about the complex temporal dynamics of psychotic symptoms and opens a window for new lines of clinical investigation [24]. Our results suggest that future researchers can reasonably expect participants to respond to two-thirds of responses, and interact around 4 times per day.

The majority of studies reviewed focused more on symptom monitoring rather than treatment interventions. Although Ben-Zeev et al’s study [27] did present promising efficacy data, there remains an overall lack of evidence regarding the efficacy of these smartphone interventions. Smartphone apps for psychiatry present many unique opportunities, because therapeutic treatments for schizophrenia such as peer support, cognitive behavioral therapy, skills development could potentially be delivered via a smartphone device. However, whether such therapies would remain efficacious after been translated onto smartphone platforms is largely unknown at this time and will be an important area of future research.

### Limitations

Our study presents several limitations that must be taken into account. First, despite our comprehensive search strategy and broad inclusion criteria, it is possible that other studies of smartphone apps for patients with schizophrenia are still in progress, unpublished, or even being conducted by private sector companies outside of academia, and thus are unavailable for review. Second, there was substantial heterogeneity between studies in terms of the types of apps used and their aims, thus making it difficult to draw overarching conclusions. However, because our review found high feasibility across all studies (despite these differences in design), the overall findings do indicate that smartphone apps can be feasible for use in schizophrenia across a wide variety of contexts. For example, high user engagement was observed in both apps that used automated prompts [23,30] and those which relied mostly on self-initiated usage [27,28]. Similarly, the apps proved feasible for a broad spectrum of uses, including monitoring symptoms [23,25], promoting recovery [27,30] and even improving physical health [25,27]. A final challenge of this data is extrapolating results regarding adherence and retention over longer durations than the study periods. It is possible that participants were more adherent during the study, and would have used the apps less over time. Conversely, participants equally may have built stronger habits of regular usage over time, after finding the apps engaging and beneficial from the outset. Understanding if and how patients will use smartphone apps for months or even years to monitor and manage their own condition is a clinically important question, which future research must aim to address.

### Conclusion

Although the current literature on the role of smartphones in psychotic disorders such as schizophrenia is small, results suggest high feasibility and acceptability. However, there is currently limited data on the efficacy of smartphone apps. Furthermore, the literature supports many diverse use cases. With further research and clinical innovation, smartphones have the potential to become an important tool that psychiatrists can employ in the clinical care and management of psychotic disorders.

## Appendix

Multimedia Appendix 1 Studies of smartphone apps for people with schizophrenia.

## Abbreviations

CDS: Calgary Depression Scale
PANSS: Positive and Negative Syndrome Scale
