# Supplementary material for: Smartphone Apps for Schizophrenia: A Systematic Review
Source: JMIR Mhealth Uhealth. 2015 Nov 6;3(4):e102. doi: 10.2196/mhealth.4930 (PMC4704940; doi:10.2196/mhealth.4930)
Supplement: Multimedia Appendix 1 [file mhealth_v3i4e102_app1.pdf]

Studies	Intervention summary	Duration (days)	Total (n)	Attrition	Adherence	User experience	Reported benefits
Ainsworth et al [25]	*Android app "ClinTouch":* a randomized control trial comparing app-based vs short messaging service-based mobile phone ambulatory monitoring systems for schizophrenia.	6	24 (24 with schizophrenia)	0/24	69% of all possible entries were completed<br><br>2.8 uses per day (mean average)	The app was rated as "pleasing" overall (scoring 3.7 on a 7-point scale). The app was not rated as "stressful" or "challenging" (scoring only 1.8 and 2.2 on 7-point scales)	Participants felt the app could help them or other service users (5.3 on a 7-point scale)
Ben Zeev et al [27]	*Android app "FOCUS":* Single-arm feasibility trial of real-time/real-place illness management support for schizophrenia.	28	33 (33 with schizophrenia)	1/33 due to losing phone	Participants used FOCUS on 86.5% of days in the study<br><br>5.2 uses per day (mean average)	93.7% of participants satisfied with overall ease of use. Less than 20% found the app to be "awkward," "complicated," or "inconsistent."	87.5% of participants felt that the app helped to manage symptoms. Paired samples *t* tests showed significant reductions in positive and negative symptoms and depression.
Macias et al [30]	*iPhone and Android app 'WellWave'*: Assessing feasibility of an app which promoted walking as a physical exercise, and offered a variety of supportive nonphysical activities, including confidential text	28	11 (4 with schizophrenia)	1/11 withdrew of own accord	Used on 94% of days<br><br>73 % response rate to prompts (3.54 per day)<br><br>70% of participants	100% of participants were satisfied with the app overall. Only criticisms were made, pertaining to color/sound	Participants experienced both improved well-being (eg, put my head in a good place) and practical

	messaging with peer staff and a digital library of videos on recovery.				achieved ≥2 walks per week	preferences, and the study coming to an end.	benefits (eg, Motivated me to get up and walk around the block).
Naslund et al [28]<br><br>Aschbrenner et al [29]	*iPhone app "PeerFIT" with wearable activity monitor*: Assessing feasibility and acceptability of popular m-health technologies for activity tracking among overweight and obese individuals with serious mental illness	80-133	10 (3 with schizophrenia)	1/10 withdrew due to medical reasons	Participants used activity monitors on 89% of days in the study	100% were "very satisfied" or "somewhat satisfied" with PeerFIT overall; 60% would recommend to a friend. Participants felt the devices were expensive for low-income individuals.	100% found the program helped them to reach their goals. Mean weight loss of 2.7 kg across all participants (P>.05).
Palmier-Claus et al [23,24]	*Android app "ClinTouch":* Single-arm feasibility trial of an app-based monitoring system for psychosis.	7	44 (36 with schizophrenia)	8/44 due to noncompliance	72% of all possible entries were completed<br><br>4.4 uses per day (mean)	Not reported	Smartphone app provided clinically valid real-time measures of psychotic symptoms and affective state
